# Automated wound segmentation and classification of seven common injuries in forensic medicine

**DOI:** 10.1007/s12024-023-00668-5

**Published:** 2023-06-28

**Authors:** Norio Zimmermann, Till Sieberth, Akos Dobay

**Affiliations:** 1 Zurich Institute of Forensic Medicine, University of Zurich, Winterthurerstrasse 190/52, CH-8057 Zurich, Switzerland; 2 3D Centre Zurich, University of Zurich, Winterthurerstrasse 190/52, CH-8057 Zurich, Switzerland; 3 Forensic Machine Learning Technology Center, University of Zurich, Winterthurerstrasse 190/52, CH-8057 Zurich, Switzerland

**Keywords:** Deep learning, Forensic sciences, Image segmentation, Wound classification

## Abstract

In forensic medical investigations, physical injuries are documented with photographs accompanied by written reports. Automatic segmentation and classification of wounds on these photographs could provide forensic pathologists with a tool to improve the assessment of injuries and accelerate the reporting process. In this pilot study, we trained and compared several preexisting deep learning architectures for image segmentation and wound classification on forensically relevant photographs in our database. The best scores were a mean pixel accuracy of 69.4% and a mean intersection over union (IoU) of 48.6% when evaluating the trained models on our test set. The models had difficulty distinguishing the background from wounded areas. As an example, image pixels showing subcutaneous hematomas or skin abrasions were assigned to the background class in 31% of cases. Stab wounds, on the other hand, were reliably classified with a pixel accuracy of 93%. These results can be partially attributed to undefined wound boundaries for some types of injuries, such as subcutaneous hematoma. However, despite the large class imbalance, we demonstrate that the best trained models could reliably distinguish among seven of the most common wounds encountered in forensic medical investigations.

**Supplementary Information:**

The online version contains supplementary material available at 10.1007/s12024-023-00668-5.

## Introduction

In this pilot study, our aim was to automatically segment and classify skin wounds on photographs taken during forensic medical examinations of injured persons. Performing segmentation prior to the assessment by a forensic pathologist could reduce the time needed for examination and documentation. Our approach of using neural networks for image-based wound assessments in a legal or investigative context is not yet fully covered in the literature.

Anisuzzaman et al. (2020) discuss three main algorithms for image-based wound assessment: rule-based, machine learning, and deep learning—a subset of machine learning based on artificial neural networks [1]. Rule-based algorithms rely on manual wound descriptions [2, 3], while machine learning and deep learning algorithms can be trained to extract features directly from the images and use this information for further assessments. For example, Li et al. (2018) employed a composite model that combines watershed segmentation with dynamic thresholding and deep learning [4].

In recent years, several papers on image-based wound assessment have been published. However, unlike our study, most of the papers focus on chronic wounds and not forensic images [1, 4–12]. Wang et al. (2020) built a deep learning framework for the automatic segmentation of chronic wounds [7]. Other related works examined burn marks [13–16]. For example, Jiao et al. (2019) proposed a deep learning method based on mask region-based convolutional neural networks, which generate regions of interest on the images and then detect and segment burn wounds from each of those regions [13]. In forensics, Oura et al. (2021) utilized artificial neural networks to assess the shooting distance using images of gunshot wounds in piglets [17].

Among the most successful and commonly employed techniques for image segmentation are convolutional neural networks (CNNs) [18–20]. Many models currently use an encoder-decoder architecture in their CNN [19]. The encoder downsamples the input image to extract features, while the decoder upsamples the feature maps to gradually recover the full resolution of the input image necessary to establish a segmentation map. Skipped connections—a technique where some of the hidden layers in the neural network are skipped in an alternative path—are often used to combine coarse-grained, high-level information with fine-grained, low-level information [19]. As an example, in Wang et al. (2015), the authors developed a simple encoder-decoder CNN architecture to segment wound regions [5]. Feature pyramid network (FPN) architectures are based on a pyramidal hierarchy of CNNs that consist of a bottom-up pathway and a top-down pathway. The bottom-up feature encoder is connected via lateral connections to the top-down pathway, which were developed for building high-level semantic feature maps at various scales [21]. Another popular architecture for medical image segmentation is U-Net, which also builds on an encoder-decoder architecture [22].

A variety of networks can serve as encoders. For example, ResNet uses skip connections to facilitate the training of deep neural networks [23]. ResNeXt extends this technique by splitting the input of each building block into branches, transforming them, and then merging them again [24]. Squeeze-and-excitation (SE) networks, for example, SE-ResNeXt, add a channelwise attention mechanism to the CNN architecture [25]. ResNeSt also builds on the ResNeXt architecture by applying channelwise split attention on the different network branches [26]. EfficientNet builds on mobile inverted bottleneck blocks combined with squeeze-and-excitation layers, and a compound scaling factor was used to rescale the models to the desired size [27].

We compare different existing encoder-decoder architectures and loss functions for the segmentation and classification of wounds on forensic images.

## Methods

For this study, we labeled photographs from our forensic photo database to train preexisting CNNs and compare their suitability for wound segmentation and classification on our test dataset. Information about data augmentation, data preprocessing, and evaluation metrics is provided in Online Resource 1.

### Case selection

**Fig. 1:**
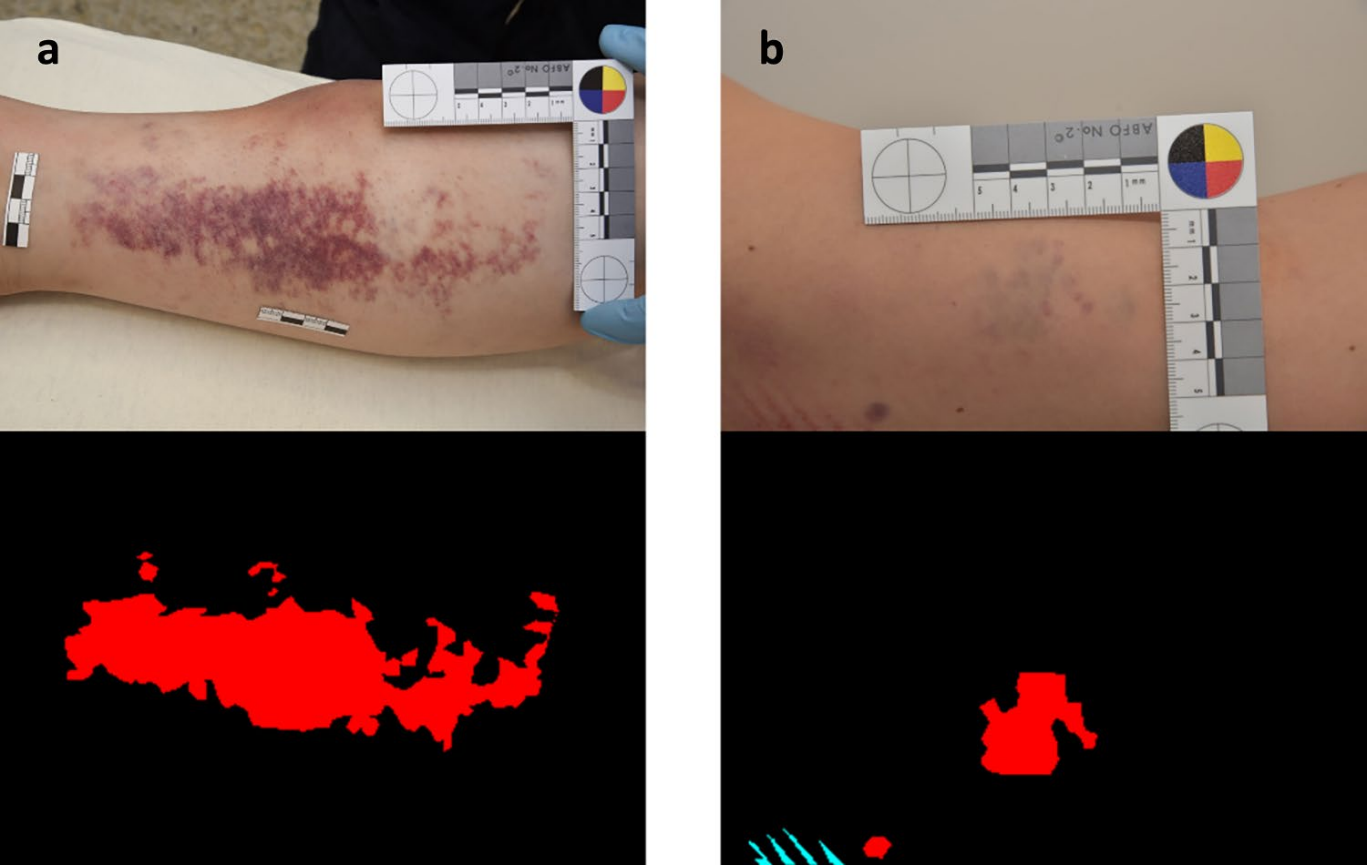
Photographs showing two examples of wounds from the training set. The picture on the left (**a**) is an example of small potentially nonwounded and unspecified areas that have been classified as subcutaneous hematomas. In this case, it is unclear whether this wound should be classified as one or several wounds. The picture on the right (**b**) is an example of a subcutaneous hematoma where the exact boundaries are not clearly defined and parts are barely visible (red, subcutaneous hematoma; blue, skin abrasion)

Wound images were retrospectively extracted from our database. We included all closed cases from January 2017 to September 2020 that had a clinical forensic medical examination performed at the Zurich Institute of Forensic Medicine. A total of 1753 pictures from 817 cases were extracted, showing 4666 separate wounded regions. During the extraction, we ensured that at least one skin injury was visible in each photograph. There were no exclusion criteria. However, if several photographs of a specific wound were available, usually only one photograph was chosen to keep the diversity as high as possible. We also included photographs of untreated wounds taken by the staff from hospitals where the injured person was treated. We deliberately included images containing small or barely visible lesions and some treated wounds, over- or underexposed photographs, images with blurry wounds in the background, and images with tattoos next to the wounds. These photographs should strengthen the models to become more resilient against generating incorrect predictions from poor-quality images (Fig. 1b).

### Dataset

Semantic wound segmentation and classification were realized by a medical student with the *VGG Image Annotator* (VIA*) version 2.0.10* using the forensic reports as a reference [28]. Semantic image segmentation describes the task of assigning each pixel of the input image to a certain class [18–20]. Instance image segmentation extends this approach by delineating and detecting individual objects as separate objects, even if they belong to the same class [19]. We chose semantic segmentation for our study; for example, skin abrasions and subcutaneous hematomas could sometimes be classified as a single wound or as a collection of wounds within one area (Fig. 1a). Another difficulty when segmenting images is that the wound boundaries are often not well delineated. For example, different experts might place the boundaries of a subcutaneous hematoma differently when looking at the same image (Fig. 1b).

**Fig. 2:**
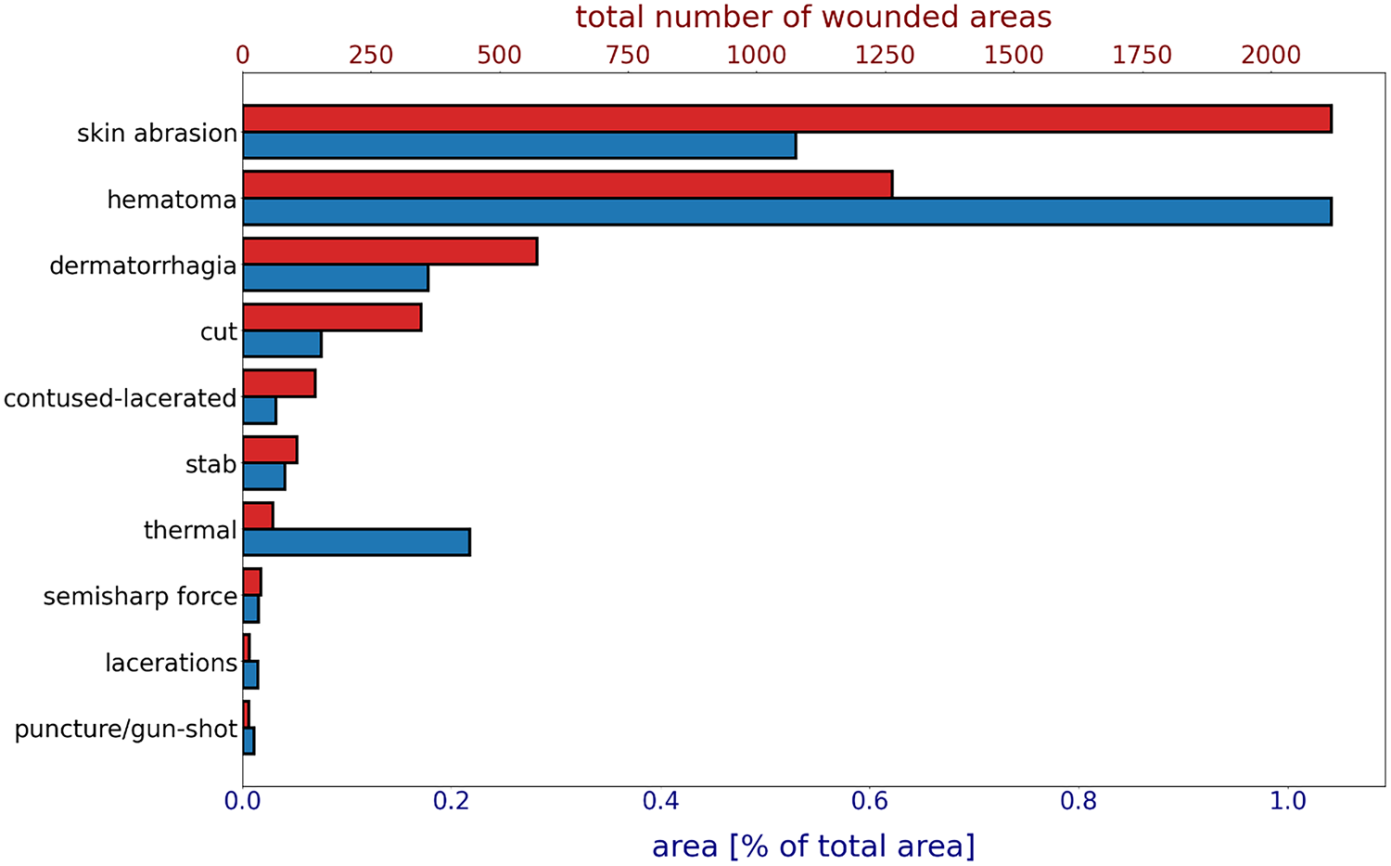
Histogram showing the total number of wounds (red) for each category and the ratio of wound to image surface area expressed as a percentage (blue)

All images were rechecked to avoid classification mistakes. Cases with unclear classification were discussed with a board-certified forensic pathologist. The wounds were labeled using ten different categories: (1) skin abrasion, (2) subcutaneous hematoma (bleeding under the skin), (3) dermatorrhagia (bleeding into the skin), (4) cut, (5) contused-lacerated, (6) stab, (7) thermal, (8) semisharp force, (9) puncture/gunshot wound, and (10) laceration. Figure 2 presents the total number of wounds per category (red bars) and shows the ratio of the wound area to the total image area in percentage and per category (blue bars). We subsequently removed wounds in categories (8) semisharp force, (9) puncture/gunshot wound, and (10) laceration from the results, as we realized that their numbers were too few for a proper model comparison.

As shown in Fig. 2, there was a large wound area-to-image area imbalance. Even the most prominent class of injury, subcutaneous hematoma, has only approximately 1% of all image pixels assigned to it, and all wounds combined comprised only approximately 2%. Additionally, there was a very significant imbalance among the different wound classes for both total wound area and wound counts.

Some wounds, such as bleeding into the skin (mostly petechiae) or abrasions, often consist of dozens of small injuries in one area that are hard to individually segment. In these cases, sometimes, the whole area was classified as one wound, even though there was some healthy skin near the injuries (Fig. 1a). In some rarer cases, it was possible to have two correct wound class labels in the same injured area. These regions were assigned to the visually more dominant class. For example, if an area showed clear superficial skin abrasions with a barely visible subcutaneous hematoma underneath, then the wound was labeled as skin abrasion.

Not all injuries could be reliably classified. To address this label noise, a likelihood attribute for each wounded region was included in the data by a medical student. The likelihood corresponded to “not certain,” “quite certain,” or “very certain.”

### Training

To evaluate our models, we randomly selected a test set of 182 images. We applied sevenfold cross-validation from *scikit-learn* [29] to randomly split the remaining data into seven groups. In each fold, one of these groups served as the validation set with 225 images, and the other 6 groups served as the training set with 1346 images.

During training, we used a batch size of eight images. All training images were randomly shuffled between each epoch for a total of 100 epochs. The model parameters were updated using the *Adam optimizer algorithm* [30]. The initial learning rate, which determines the step size toward the minimum of the loss function, was set to $1\times {10}^{-4}$ and then reduced by multiplying it by a factor of 0.98 after each epoch, reducing it to approximately $1.3\times {10}^{-5}$ after 100 epochs. The training pipeline was implemented using PyTorch [31]. The models with the best mean pixel accuracy on the validation set and the models that had been trained for 100 epochs were saved and later evaluated. All results were averaged sevenfold.

### Loss function

Commonly employed loss functions for image segmentation include the pixelwise binary cross-entropy (BCE) loss and Dice loss functions. The Dice loss can be generalized to obtain the Tversky loss function, which enables us to control both the number of false-positives and the number of false-negatives with two parameters $\alpha$ and $\beta$. The focal Tversky loss (FTL) function introduced by Abraham and Khan additionally allows us to change the contributions of the different classes with respect to the Tversky loss function by adding a $\gamma$ factor to the function [32]. We chose $\alpha$ = 0.7, $\beta$ = 0.3, and $\gamma$ = 1.3 as Abraham and Khan reported good results using these values in their paper [32].

We tested different combinations of the focal Tversky and BCE loss functions. However, the weighted BCE loss function as described in Eq. (1) yielded the best results. The weights ${w}_{c}$, which were chosen inversely proportional to the label frequencies (wound areas) ${f}_{c}$, allowed us to counteract the class imbalances. The parameter ${p}_{ic}$ represents the predicted value for a pixel, and ${g}_{ic}$ represents the ground truth. The value $N$ denotes the total number of pixels in the image. The total number of wound classes is indicated by $c$. The smoothing factor $\epsilon$ was set to $1\times {10}^{-3}$ to avoid extreme gradients. Additional weights ${m}_{ic}$ for each pixel were employed to take into account the certainty of classification during labeling (see the “Dataset” section for more information). The value ${m}_{ic}$ was set to 1; if the wound classification was considered very certain, ${m}_{ic}$ was set to 1.5 for that wounded area, and if it was considered not certain, ${m}_{ic}$ was set to 0.5.

1 $$\begin{aligned}\mathrm{BCE}=&-\frac{1}{c}\sum_{c}\frac{{w}_{c}}{N}{\sum }_{i=1}^{N}{{m}_{ic} g}_{ic}\,\mathrm{ log}({p}_{ic}+\epsilon )\\&+\frac{1}{N}{\sum }_{i=1}^{N}{m}_{ic} {(1-g}_{ic})\,\mathrm{ log}(1-{p}_{ic}+\epsilon )\end{aligned}$$

For our models, we initially chose weights ${w}_{c}=1/100{f}_{c}$. We also tried ${w}_{c}=\sqrt{1/100{f}_{c}}$. The constant 1/100 was chosen because subcutaneous hematoma is the most common class and covers, on average, approximately 1% of the image surface area, which leads to ${w}_{\mathrm{hematoma}}\approx 1$ and all other weights being greater than 1. Taking the square root thus leads to lower weights. The weights exceeded one, as only the first term in Eq. (1) is weighted with ${w}_{c}$. Consequently, the first term is weighted higher than the second term, and the incentive for the model to avoid false-negative predictions is higher than that to avoid false-positive predictions.

### Model selection

Initial tests were carried out with a combination of FPN and U-Net decoders and four different encoders: EfficientNet-B3, ResNeSt-50, ResNet-50, and SE-ResNeXt-50. They were trained with a weighted (${w}_{c}=1/100{f}_{c}$) BCE loss function without using ${m}_{ic}$ weights according to the certainty of classification (see the “Loss function” section for more details). We downloaded the encoders and the FPN and U-Net decoders from *Segmentation Models PyTorch—v0.1.2* [33] and used pretrained ImageNet weights for transfer learning. The default numbers of layers and filters were left unchanged. A softmax2d activation function provided the final predictions, after which each pixel was assigned to the class with the highest prediction. SE-ResNeXt-50 U-Net showed the best results in this first round. Further tests focused on different loss functions and weights, which were compared by training the SE-ResNeXt-50 U-Net and SE-ResNeXt-50 FPN models to determine if changes in the loss function equally affect both model architectures.

## Results

In this study, we compared different encoder-decoder architectures and loss functions based on the mean IoU and mean pixel accuracy. All results were averaged sevenfold. Online Resource 2 gives an overview of the results for different combinations of encoders and decoders on the test dataset. The best scores were achieved by SE-ResNeXt-50 in combination with a U-Net decoder. The model with the best mean validation accuracy was evaluated on the test dataset. Averaged sevenfold, the model achieved a mean pixel accuracy of 67.5% and a mean IoU of 39.9% on the test set. The other encoders gave superior results in combination with an FPN compared to a U-Net decoder. Consequently, the mean pixel accuracy was 3.4%, and the mean IoU was 3.6% higher on average for the FPN.

As shown in Online Resource 2, the models with the best mean pixel accuracy evaluated on the validation set have a better mean pixel accuracy on the test set than the models trained for the full 100 epochs. For the 8 models in Online Resource 2, the decrease in mean pixel accuracy was approximately 3.1% on average. On the other hand, the mean IoU was on average 6.2% higher after 100 completed epochs of training.

**Fig. 3:**
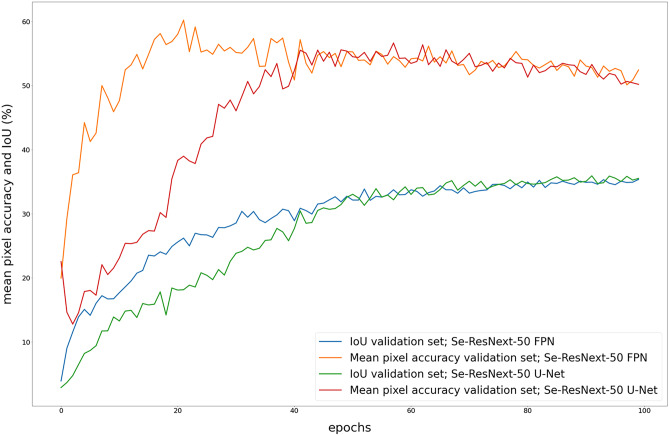
Time series plot showing the IoU and mean pixel accuracy for each epoch, averaged sevenfold and evaluated on the validation sets. The U-Net architecture appears to learn at a slower rate than the FPN architecture. While the IoU continues to improve up to the last epoch, the mean pixel accuracy peaks between epoch 20 and epoch 60 and then continues to slightly decrease

Figure 3 shows the mean pixel accuracy and IoU for SE-ResNeXt-50 U-Net and SE-ResNeXt-50 FPN evaluated on the validation sets for each epoch. We observed that the mean pixel accuracy peaks after 20 to 60 epochs and shows a minimal downward tendency. In comparison, the IoU continues to slightly improve up to epoch 100. Generally, we observed that FPN architectures learned at a faster rate than U-Net architectures.

Online Resource 3 shows an overview of different combinations of the focal Tversky and binary cross-entropy loss functions, as well as the effects of using different weights. All loss functions were tested with SE-ResNeXt-50 FPN and SE-ResNeXt-50 U-Net. Overall, the SE-ResNeXt-50 U-Net and FPN architectures were equally good, with U-Net having a 0.7% higher mean pixel accuracy but a mean IoU that was 0.6% lower than the FPN on average.

The BCE loss function showed results superior to the FTL and combinations of FTL and BCE (Online Resource 3).

In our baseline BCE loss function (Eq. 1), only the first term is weighted. Weighting the second term also led to a 9.9% lower mean pixel accuracy but a 2.7% increase in the mean IoU on average. Using smaller class weights by taking the square root of the weights (${w}_{c}=\sqrt{1/100{f}_{c}}$) led to a 1.2% decrease in the mean pixel accuracy but a 4.2% increase on average in the mean IoU for all loss functions compared to the original weights ${w}_{c}= 1/100{f}_{c}$.

Our dataset included information regarding the level of classification certainty during the labeling of the wounds, which was utilized in the loss function by adding additional weights (for more details, see the “Dataset” and “Loss function” sections). With the added weights, we achieved the highest mean pixel accuracy of 69.4% and a mean IoU of 38.1% with SE-ResNeXt-50 FPN. The additional weights increased the mean pixel accuracy by 1.9% on average. The effects on the mean IoU were variable, with an average decrease of 1.9% for U-Net but an increase of 0.9% for FPN. Combining this loss with smaller class weights by taking the square root of the weights $({w}_{c}=\sqrt{1/100{f}_{c}})$, we achieved the highest observed mean IoU of 48.7% but a lower pixel accuracy of 63.4% again with the SE-ResNeXt-50 FPN.

**Fig. 4:**
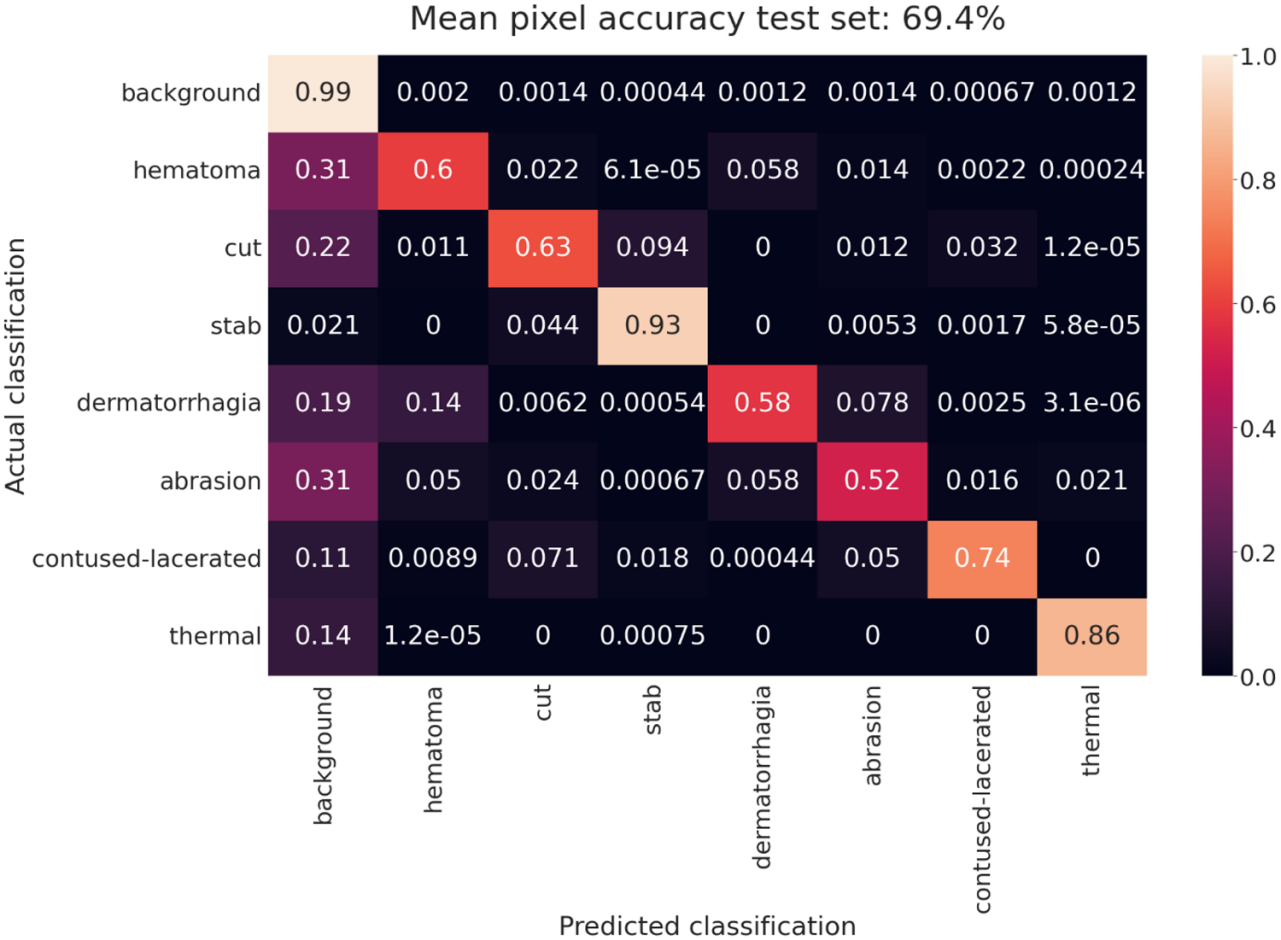
Confusion matrix showing the mean pixel accuracy evaluated on the test set averaged sevenfold. The selected model is SE-ResNeXt-50 FPN, trained with a BCE loss function with ${w}_{c}=1/100{f}_{c}$ and weights according to the certainty of classification (Eq. 1). Then, the model with the best mean pixel accuracy on the validation sets was selected. All results are averaged sevenfold

The confusion matrix in Fig. 4 shows the pixel accuracies evaluated on the test set for the model with the highest mean pixel accuracy, as described above. The model fails to fully distinguish wounds from the background. In particular, pixels classified as subcutaneous hematomas and skin abrasions were incorrectly assigned to the background class in approximately 31% of cases. The model is also unsuccessful in fully differentiating dermatorrhagia and subcutaneous hematoma, with 14% incorrectly classified as the latter. Stab wounds, however, were very reliably classified with a pixel accuracy of approximately 93% in the test dataset, and the network quite successfully distinguished cut and stab wounds, with less than 10% of cut pixels being classified as stab wounds and less than 5% of stab wound pixels being classified as cuts.

## Discussion

The main goal of our study was to train a reliable algorithm to automate forensic wound segmentation and classification. Related studies assessed relatively large wounds with well-defined borders and few classes [4–9, 13]. This choice makes it difficult to directly compare their results with our results. More importantly, most studies focus on chronic wounds and not forensic images [1, 4–12]. Forensic images usually show more contextual information and different wound classes. Furthermore, the photographs often have a higher resolution and several types of wounds on the same image.

We assume that the high overlap among subcutaneous hematoma, skin abrasion, and background depicted in Fig. 4 can be partially explained by often undefined wound boundaries for these classes. Moreover, we observed a tradeoff between IoU and pixel accuracy. While the mean pixel accuracy peaks early, the IoU continues to improve (Fig. 3). We conclude that the optimal number of epochs depends on whether the aim is to reach the highest possible IoU or the highest pixel accuracy.

Similarly, reducing the weights by taking the square root of the weights increased the mean IoU, but only at the cost of a lower mean pixel accuracy. One explanation for this effect is that smaller loss weights led to a smaller image area assigned to the wound classes, resulting in substantially fewer false-positive predictions and a better IoU. There was also a slight increase in false-negatives and a decrease in true positives, which caused a decrease in the pixel accuracy. We observed substantially better results with the BCE loss function than with the FTL (Online Resource 3). The large difference might be partially attributed to the parameters $\alpha$, $\beta$, and γ used in the FTL, which potentially could be further optimized for our models and dataset.

Collecting additional data to improve the certainty of classification during the manual segmentation and classification of the wounds helped us further increase the mean pixel accuracy. Compared to the segmentation of the wounds, only a small additional effort was needed to collect these data. Based on our results, using this information seems worthwhile in the case of datasets with a significant proportion of wounds that are difficult to classify with high certainty just by looking at the photographs.

**Fig. 5:**
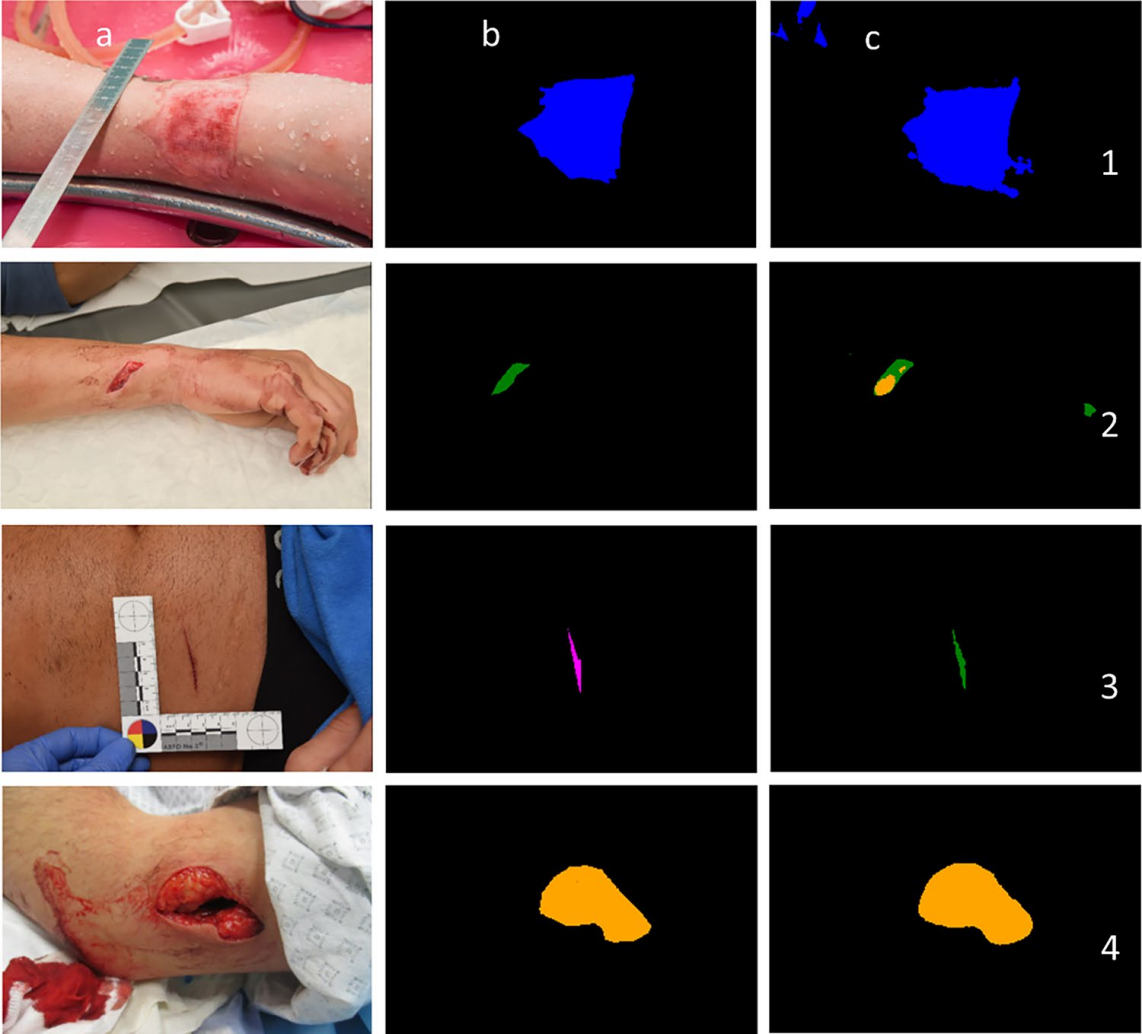
Illustration of photographs with their corresponding predictions made by the SE-ResNeXt-50 FPN model on our test dataset. The network was trained with a BCE loss function, ${w}_{c}=1/100{f}_{c}$, and weights according to the certainty of classification (Eq. 1). Then, the model with the best mean pixel accuracy on the validation sets was selected. Columns from left to right: input image (**a**), ground truth/target label (**b**), and its predictions (**c**). The first three rows depict common mistakes by the network: mistaking parts of the background as wounds and other obvious mistakes [1]; center of cut classified as stab wounds and dried blood classified as a cut and other understandable mistakes [2]; misclassification of skin abrasion as a cut wound [3]. Using only the photograph, especially at a low resolution, it is difficult for a human to determine the correct classification. Row four depicts an additional example from our test set [4]. The model correctly classifies the blood on the cloth and arm as background. (blue, thermal wound; green, cut; orange, stab wound; and magenta, skin abrasion)

Figure 5 shows a collection of true and false predictions obtained on the test set. For instance, mistakes were made by the network that would be obvious to a human, such as mistaking parts of the background as wounds (Fig. 5 (1)). Increasing the size and diversity of our dataset could avoid this kind of mistake. One of the limitations to this approach is the low number of newly closed cases and the time needed to mask the images. Another type of less obvious mistake is visible in Fig. 5 (2). The network correctly classified parts of the cut. However, the wider center part of the wound was labeled stab. Additionally, dried blood on the hand was classified as a cut. The third type of misclassification is hard to assess even for a human without zooming in on a photograph or having additional background information on the case. An example is Fig. 5 (3), where the model predicts a cut, while the ground truth classification is skin abrasion. At a lower resolution, it is difficult, even for a human eye, to confidently assess the type of wound. Such errors are quite rare and might be fixed by increasing the resolution of the training images. In our study, this size was set to $512\times 512$ pixels due to limited computing resources. Figure 5 (4) depicts an additional example from our test set. Note that the model correctly classifies the blood on the cloth and arm as background.

Preventing the abovementioned misclassifications and thus reducing false-positive and false-negative predictions remain challenging. In praxis, a photograph is not always sufficient to correctly classify a wound. Currently, human supervision is still critical for correcting false predictions.

Future studies could test the networks with other methods employed in medical forensic imaging, such as three-dimensional photogrammetry [34]. Future studies might also expand the network with multilabel classification in the case of overlapping wounds and add a postprocessing step to remove very small wound predictions that would otherwise cost substantial time to revise if incorrect. However, the next step will be to use wound segmentations and classifications to automatically create short wound descriptions and thus facilitate the documentation process.

## Key points

1. SE-ResNeXt-50 encoders combined with FPN or U-Net decoder models are suitable for differentiating wounds on photographs.
2. Most common errors are linked to misclassifying backgrounds as wounds and vice versa due to undefined wound boundaries.
3. Stab wounds were reliably classified with a pixel accuracy of 93%.
4. Use of a weighted BCE loss function gave the best results.

### Supplementary Information

Below is the link to the electronic supplementary material.

Supplementary file1 (DOCX 38 KB)

Supplementary file2 (DOCX 15 KB)

Supplementary file3 (DOCX 16 KB)

## Data Availability

The datasets analyzed during the current study are not publicly available due to data privacy. The code and the trained models are available on reasonable request.
