# Supplementary material for: Automated wound segmentation and classification of seven common injuries in forensic medicine
Source: Forensic Sci Med Pathol. 2023 Jun 28;20(2):443–51. doi: 10.1007/s12024-023-00668-5 (PMC11297066; doi:10.1007/s12024-023-00668-5)
Supplement: Supplementary file 1 — Supplementary file1 (DOCX 38 KB) [file 12024_2023_668_MOESM1_ESM.docx]

**Information about data augmentation, preprocessing, evaluation metrics, and hardware requirements**

**Data augmentation and preprocessing**

Because most of the images were $6000\times4000$ (3:2) pixels in size, we rescaled them using a bilinear interpolation function from *OpenCV – 4.0.1* [1]. The smaller side was set to 512 pixels, while the larger side was proportionally rescaled. To increase the diversity among the wounds, we also applied image augmentation techniques using *Kornia* during the training phase [2]. All the images were randomly shifted, rescaled, and rotated. The images were also randomly flipped both horizontally and vertically. All the images were cropped at a randomly chosen position to a fixed size of $512\times512$ pixels.

In addition, we preprocessed our image data with RGB normalization using the mean and standard deviation values as a rescaling method.

**Evaluation metrics**

To evaluate the precision of our classifiers, we determined the mean intersection over union (IoU) and the mean pixel accuracy of all labels, excluding the background. To address the large class imbalance and prevent the most common classes from dominating the results, the metrics were individually calculated for each class and then averaged. The wounds were equally weighted despite significant class imbalances to increase the influence of rarer wound classes on the metrics. The pixel accuracy was calculated by dividing the true-positive predictions of a class, that is, the number of image pixels correctly assigned to that wound, by the sum of true-positive and false-negative predictions of that class:

$Pixel accuracy= \frac{True positives}{True positives + False negatives}$

The IoU, on the other hand, was calculated by dividing the number of true-positive predictions by the sum of false-positive, false-negative, and true-positive predictions:

$Intersection over Union= \frac{True positives}{True positives + False negatives + False positives}$

It follows that the pixel accuracy of the model is not influenced by false-positive predictions but allows us to assess how well a model can distinguish among different classes. On the other hand, the IoU is influenced by both false positives and false negatives.

**Hardware requirements**

The data augmentation and model training were performed on graphics processing units (NVIDIA RTX 2080 Titan) with approximately 11 GB GDDR6 memory.

References

1. Culjak I, Abram D, Pribanic T, Hrvoje Dzapo, Cifrek Mario. A brief introduction to OpenCV. Proceedings of the 35th International Convention MIPRO. 2012:1725–30.

2. Riba E, Mishkin D, Ponsa D, Rublee E, Bradski G. Kornia: an Open Source Differentiable Computer Vision Library for PyTorch; 05.10.2019.
