# Supplementary material for: Automated wound segmentation and classification of seven common injuries in forensic medicine
Source: Forensic Sci Med Pathol. 2023 Jun 28;20(2):443–51. doi: 10.1007/s12024-023-00668-5 (PMC11297066; doi:10.1007/s12024-023-00668-5)
Supplement: Supplementary file 2 — Supplementary file2 (DOCX 15 KB) [file 12024_2023_668_MOESM2_ESM.docx]

|  | mean pixel accuracy test set | mean iou test set | mean pixel accuracy test set (last epoch) | mean iou test set (last epoch) |
| --- | --- | --- | --- | --- |
| Se-ResNeXt-50-FPN | 66.24% | 31.30% | 61.22% | 44.00% |
| Efficientnet-B3-FPN | 64.86% | 36.03% | 63.96% | 42.72% |
| Resnest-FPN | 67.28% | 33.33% | 62.05% | 42.40% |
| Resnet-FPN | 65.36% | 32.43% | **64.02%** | 40.26% |
| Se-ResNeXt-50-Unet | **67.53%** | **39.87%** | 61.51% | **44.74%** |
| Efficientnet-B3-Unet | 60.26% | 27.97% | 59.24% | 31.53% |
| Resnest-Unet | 64.63% | 35.31% | 61.55% | 37.59% |
| Resnet-Unet | 57.67% | 27.04% | 55.30% | 30.00% |

**Table 1 Overview of the mean intersection over union (IoU) and mean pixel accuracies for different model architectures**

All models were trained with a weighted BCE loss ($w_{c}=1/100f_{c}$) without using weights $m_{ic}$ according to the certainty of classification (see Equation 1 in the main text). The two columns on the left show the models with the best mean pixel accuracies on the validation sets. The columns on the right show the models trained for 100 epochs. The metrics were evaluated on the test set and averaged over seven folds. The best results overall were achieved with the SE-ResNeXt-50 U-Net model. The numbers in bold denote the best results.
