# Supplementary material for: Automated wound segmentation and classification of seven common injuries in forensic medicine
Source: Forensic Sci Med Pathol. 2023 Jun 28;20(2):443–51. doi: 10.1007/s12024-023-00668-5 (PMC11297066; doi:10.1007/s12024-023-00668-5)
Supplement: Supplementary file 3 — Supplementary file3 (DOCX 16 KB) [file 12024_2023_668_MOESM3_ESM.docx]

|  | mean pixel accuracy test set | mean iou test set | mean pixel accuracy test set (last epoch) | mean iou test set (last epoch) |
| --- | --- | --- | --- | --- |
| Se-ResNeXt-50-FPN  BCE | 66.24% | 31.30% | 61.22% | 44.00% |
| Se-ResNeXt-50-UNET  BCE | 67.53% | 39.87% | 61.51% | 44.74% |
| Se-ResNeXt-50-FPN  0.5*BCE + 0.5*FTL | 62.58% | 33.76% | 57.38% | 38.07% |
| Se-ResNeXt-50- UNET  0.5*BCE + 0.5*FTL | 59.51% | 31.48% | 59.53% | 32.53% |
| Se-ResNeXt-50-FPN  FTL | 51.27% | 25.99% | 48.55% | 26.94% |
| Se-ResNeXt-50- UNET  FTL | 58.92% | 25.03% | 57.26% | 25.60% |
| Se-ResNeXt-50-FPN  BCE (2 weights) | 57.35% | 41.80% | 52.87% | 44.62% |
| Se-ResNeXt-50-UNET  BCE (2 WEIGHTS) | 53.89% | 41.67% | 52.68% | 42.63% |
| Se-ResNeXt-50-FPN  BCE (sqrt weights) | 65.58% | 42.98% | 61.46% | 47.57% |
| Se-ResNeXt-50- UNET  BCE (sqrt weights) | 65.48% | **44.00%** | 59.24% | 44.85% |
| Se-ResNeXt-50-FPN  BCE-certainty | **69.40%** | 38.05% | **64.08%** | 44.16% |
| Se-ResNeXt-50- UNET  BCE-Certainty | 67.60% | 35.99% | 62.98% | 42.64% |
| Se-ResNeXt-50-FPN  BCE-certainty (sqrt weights) | 65.55% | 38.62% | 63.35% | **48.68%** |
| Se-ResNeXt-50- UNET  BCE-Certainty (sqrt weights) | 66.91% | 40.20% | 63.44% | 47.05% |

**Table 1: Overview of the mean intersection over union (IoU) and mean pixel accuracy for different loss functions and weights**

The two columns on the left show the models with the best mean pixel accuracies on the validation sets. The columns on the right show the models trained for 100 epochs. The metrics were evaluated on the test set and averaged over seven folds. Both SE-ResNeXt-50 U-Net and SE-ResNeXt-50 FPN were used to compare the different loss functions.

The indication *BCE* in the table denotes a weighted BCE loss function ($w_{c}=1/100f_{c}$), without using weights $m_{ic}$ according to the certainty of classification (see Equation 1 in the main text). 0.5**BCE + 0.5*FTL* corresponds to the combination of BCE and FTL. For the indication *FTL,* we used $\alpha$ = 0.7, $\beta$ = 0.3 and $\gamma$ = 1.3 and $w_{c}=1/100f_{c}$, the same as for the BCE loss. *BCE (sqrt weights)* denotes that the square root of the initial weights was used ($w_{c}=\sqrt{1/100f_{c}}$). The indication *BCE-certainty* stands for a BCE loss with weights $m_{ic}$ according to the certainty of classification. Finally, for *BCE-certainty (sqrt weights),* we used a combination of the previous two. The numbers in bold denote the best results.
